# Learning Verbs in Sentences: Children With Developmental Language Disorder and the Role of Retrieval Practice

**DOI:** 10.1044/2024_JSLHR-24-00321

**Published:** 2024-10-03

**Authors:** Laurence B. Leonard, Patricia Deevy, Sharon L. Christ, Jeffrey D. Karpicke, Justin B. Kueser, Kaitlyn Fischer

**Affiliations:** a Department of Speech, Language, and Hearing Sciences, Purdue University, West Lafayette, IN; b Department of Human Development and Family Science, Purdue University, West Lafayette, IN; c Department of Psychological Sciences, Purdue University, West Lafayette, IN; d Boys Town National Research Hospital, Omaha, NE

## Abstract

**Purpose:**

Retrieval practice has been shown to assist the word learning of children with developmental language disorder (DLD). Although this has been true for learning new verbs as well as new nouns and adjectives, these children's overall verb learning has remained quite low. In this preregistered study, we presented novel verbs in transitive sentences with varying subjects/agents and objects/patients to determine if recall could be improved and if retrieval practice continued to be facilitative.

**Method:**

Fourteen children with DLD aged 4–5 years and 13 same-age peers with typical language development (TD) learned eight novel verbs over two sessions. Half of the novel verbs were presented with spacing between study and retrieval trials, and half were presented with the same frequency in study trials without the opportunity for retrieval. All novel verbs were presented in sentences such as, “The woman is deeking the shoe.” Children's ability to recall and use the novel verbs in the same sentence structure was tested after the second session and 1 week later. The children were also required to use the novel verbs in bare-stem form in a new structure, as in, “That woman likes to deek the towel.”

**Results:**

Both groups of children showed increased recall relative to a previous novel verb study. The children with TD showed the expected advantages of spaced retrieval over repeated study and could use the novel verbs in the new morphological form and sentence structure. The children with DLD, however, showed an advantage for spaced retrieval only shortly after the learning period. These children had great difficulty changing the novel verbs to a bare stem and using them in a new structure.

**Conclusion:**

Although spaced retrieval assists children's novel verb recall, children with DLD in particular require additional help using these verbs with morphological and syntactic flexibility.

Word learning remains one of the most persistent challenges facing individuals with developmental language disorder (DLD). Whether assessed during the preschool years or in the adult years, the vocabularies of people with DLD fall below the levels observed in their peers (e.g., McGregor, Arbisi, & Eden, 2017; McGregor, Oleson, et al., 2013; McGregor et al., 2021; Rice & Hoffman, 2015; Storkel et al., 2017). This weakness is also evident when individuals with DLD are asked to learn novel words; they require more encounters with each word to achieve the same level of learning as their peers (e.g., Alt, 2011; Gray, 2003; Jackson et al., 2019; Kan & Windsor, 2010).

Verbs stand out as even more problematic than other aspects of vocabulary (e.g., Fletcher & Peters, 1984; Svaldi et al., 2024; Watkins et al., 1993). Overall use of verbs and the number of different verbs used by children with DLD reveal weaknesses relative to not only same-age peers but also typical children 2 years younger (e.g., Conti-Ramsden & Jones, 1997; Rice & Bode, 1993). Studies of novel verb learning show that children with DLD require more exposures than their same-age peers, although even in this case, they may not reach age expectations (Nash & Donaldson, 2005; Svaldi et al., 2024). The gap between children with DLD and their peers is even greater for learning novel verbs than for learning novel nouns (Kan & Windsor, 2010).

Some of the difficulties with verbs experienced by children with DLD are likely shared by all children. Relative to nouns, verbs cannot be individuated and are inherently relational (Gentner, 1978). They are also less imageable (Ma et al., 2009). However, for children with DLD, there are other language learning challenges that may compound their difficulty with verbs. Syntactic weaknesses probably contribute to the verb learning difficulties, such as determining which complements go with which verbs (e.g., Owen Van Horne & Lin, 2011) and what the proper argument structure is for particular verbs (e.g., Grela & Leonard, 2000). The children's inconsistency with the grammatical morphemes associated with verbs could also contribute (e.g., Bedore & Leonard, 1998; Rice & Wexler, 1996). Difficulties deciphering lexical aspect, too, appear to hamper these children's verb development. For example, unlike their peers, they do not take advantage of information provided by telic verbs regarding when past tense morphemes might be applied (e.g., Leonard & Deevy, 2010; Leonard et al., 2007).

Less obviously, verbs have prosodic and phonetic features that can also pose problems. For example, relative to nouns, verbs have less typical stress patterns, fewer syllables, and shorter durations in sentences—all relatively vulnerable areas in children with DLD (e.g., Black & Chiat, 2003).

## Retrieval Practice and Verb Learning

There have been numerous studies focused on finding procedures that will assist the verb learning of children with DLD. Leonard et al. (2023) joined these efforts by determining whether these children's verb learning could be bolstered if retrieval practice were added to the learning activities. Following a review of retrieval practice, we describe the Leonard et al. study in detail, as it has a major bearing on the study reported in this article.

Retrieval practice refers to the act of trying to recall or retrieve material throughout a study period rather than testing recall only at the end. Retrieval practice has a long history of improving recall of material over and beyond continuous study alone. The chief reason is that the act of retrieving creates new learning; it is not simply a means of assessing what has already been learned.

The most benefit from retrieval practice occurs when there are multiple retrieval opportunities during learning (Karpicke & Roediger, 2008) and when spacing occurs between retrieval events (Karpicke & Roediger, 2007). Spacing can be defined in terms of the amount of time that elapsed, or the amount of other materials that intervened between the point when the information must be retrieved and when it was last studied.

In recent years, there have been several studies of the effects of retrieval practice on the word learning of individuals with DLD—both adults (e.g., McGregor, Licandro, et al., 2013) and children (e.g., Gordon et al., 2024; Levlin et al., 2022). These studies have shown that repeated spaced retrieval (RSR) leads to better word learning than repeated study without retrieval (e.g., Leonard, Deevy, et al., 2019; Leonard, Karpicke, et al., 2019; McGregor, Gordon, et al., 2017) or retrieval that is always immediate with no spacing (Haebig et al., 2019). Children and adults with DLD do not learn as many words as their peers, although the gap between these groups may be narrower when RSR is used than when other procedures are employed (Leonard et al., 2021). The differences favoring typically developing peers are seen from the outset of learning; however, for those words that are learned by individuals with DLD, their retention of the words over time is quite stable (e.g., Gordon et al., 2021). Encoding appears to be the biggest obstacle for these individuals, not consolidation or long-term retention (e.g., Bishop & Hsu, 2015; Gordon et al., 2021; Jackson et al., 2021; McGregor, Gordon, et al., 2017).

The study by Leonard et al. (2023) followed these leads in the hope of finding similar facilitative effects when RSR focused on verb learning in particular. Eight novel verbs were presented to 4- and 5-year-old children with DLD and their age-mates with typical language development (TD). The novel verbs represented the names of video-recorded novel actions performed by actors. Actions were novel movements of the arms and legs that could not be labeled using common action terms. They represented intransitive activity verbs. No props were used.

The eight novel verbs and the accompanying video recordings were divided into two sets of four novel verbs. For each set, there were two learning sessions held on consecutive days. Half of the novel verbs were assigned to a repeated study condition, and half were assigned to an RSR condition. In the repeated study condition, children were only presented with study trials. For these trials, as the video clip was played, the child heard a repeated audio-recorded description of the event that included the novel verb, as in, “This woman likes to noke. She really likes to noke. This woman can really noke.” The novel verb always appeared in sentence-final position in bare-stem form. The novel verbs in the RSR condition had the same number of study trials as in the repeated study condition. However, retrieval opportunities were included for these novel verbs. Two of the retrieval trials on each day were immediate retrieval trials; after the child received a study trial for a novel verb, the video clip was shown again and the child was asked, for example, “Tell me about the man. The man likes to ___.” The remaining retrieval trials on both days were spaced trials. These retrieval trials appeared after three other novel verbs had intervened from the last time the to-be-retrieved novel verb had been heard in a study trial. All retrieval trials were followed by another study trial. The children heard the novel verbs in the two conditions an equal number of times.

Five minutes after the end of the second learning session and 1 week later, the children participated in a recall test of the novel verbs. This test included generalization items involving the novel action performed by a subject/agent who was not associated with the action during the learning period. At the 1-week point, the children were also administered two additional tests. One required the children to not only recall the novel verb but also use it in a present progressive context inflected with –*ing*, as in, “(The) woman is noking.” The other test required the child to point to the video clip that corresponded to each novel verb.

Both groups of children recalled more novel verbs in the RSR condition than in the repeated study condition. This was true directly after learning and 1 week later and for the generalization items as well as for the items that had been included during the learning period. For the test requiring the children's use of the novel verbs in present progressive contexts, the children with DLD were much less likely to use –*ing* with the novel verb even when they could recall the novel verb consistently. On the verb recognition (pointing) test, the children with DLD performed below the level of their peers, but no advantage for RSR was found.

Although RSR showed recall advantages for the children, the recall scores for the novel verbs were much lower than comparable scores for novel words representing nouns that used a very similar set of procedures (e.g., Leonard, Karpicke, et al., 2019). Even for the more favorable RSR condition, novel verb recall scores were 50% lower than the recall scores found for novel nouns in previous studies.

## The Present Study

It is not clear if the novel verb presentations in the Leonard et al. (2023) study were adequately representative of the grammatical contexts in which children hear and produce verbs in their everyday lives. In the present study, we employed a more typical subject–verb–object context while still maintaining the experimental control seen in the Leonard et al. investigation. In the present study, the children heard novel verbs in the sentence context *The Noun is Verb-ing the Noun*, as in, “The woman is pumming the cow,” and, in their retrieval attempts, were prompted to produce the novel verb as part of a full sentence of the same structure (e.g., “What's happening here? The ….” Child: “Woman (is) pumming the cow”). The context is clearly a verb context, and the structure is a common one.

To be sure, 4- and 5-year-old children with DLD (the age levels studied here) have difficulties with sentence structure, so retrieving novel verbs as part of a full sentence might be challenging. This disadvantage was offset in our view by two points. First, as noted by Arunachalam and Waxman (2015), children's verb learning may actually be facilitated when the verb appears in a rich semantic and syntactic context such as transitive contexts with noun objects/patients as well as noun subjects/agents. By varying both the subject/agent of the action and the object/patient being acted on, children have more distinct experiences with the novel verb than were provided by the intransitive actions used in the study by Leonard et al. (2023).

Second, the type of task used during the learning period has much in common with comprehension-to-production syntactic priming (e.g., Bock et al., 2007; Contemori, 2022). Throughout the novel verb learning period, the children hear a consistent syntactic structure *(The Noun is Verb-ing the Noun)* and are periodically prompted to produce this structure with one of the novel verbs. In syntactic priming tasks, the target structure is one that the participant can already use, although priming is stronger for less frequently used structures, including those that have been more recently acquired (Weber et al., 2019). In a separate task (see Method), the participants in the present study had demonstrated an ability to use the *(The) Noun (is) Verb-ing the Noun* structure in sentences with familiar verbs, although with only inconsistent use of the auxiliary *is*. Consistent with the implicit learning view of priming (Bock & Griffin, 2000; Chang et al., 2006), the priming that occurs during the session can be characterized as representing small incremental changes in the strength of the structure within the child's grammatical system. As applied to the present study, this holds for the repeated study condition as well as the RSR condition; according to implicit learning theory, learning primarily occurs through comprehension (e.g., Branigan & McLean, 2016; Chang et al., 2006). Indeed, priming occurs even in comprehension-to-comprehension syntactic priming where the child's response involves eye gaze (Thothathiri & Snedeker, 2008).

However, there is one notable difference between our learning task and tasks used in most priming studies. In our task, the verbs are novel and not yet established in the child's lexicon. During a retrieval trial, the child will have to access a still-fragile verb when generating the sentence.

At first blush, including shared verbs in the sentences seems to resemble the conditions that create the “lexical boost” in priming studies (Rowland et al., 2012). The lexical boost represents the added, though short-lived, strength of the priming effect when the target sentence can make use of the same open-class words—especially the verb—that appeared in the prime sentence. However, these are verbs already well established and integrated within the participant's long-term semantic and phonological memory systems. In contrast, in the present study, the retrieval of the (novel) verb that must be incorporated into the sentence is likely to be a more effortful activity.

We see the present study, then, as novel verb learning in sentences. The consistent subject–verb–object structure of the sentences should reduce the children's burden of generating the overall structure of the sentence, while the richer information of different subjects/agents and objects/patients should provide information about the novel verbs' argument structure in an engaging way.

Thus, our research questions are the following:

1.  We ask in this study if, under these learning conditions, children have more overall success than in earlier studies and if RSR can bolster learning and recall even more than similar exposure without the opportunity for retrieval. Following the learning period, the children's recall is tested not only when the novel verbs must be used in the sentences employed during the learning period but also when the novel verbs must be used in the same syntactic structure but with subjects/agents and objects/patients that were not associated with the novel verbs during the learning period.
2.  A second research question relates to the first: If novel verb learning is successful with the original (*The Noun is Verb-ing the Noun*) structure, can the children succeed in using the novel verbs when both the syntactic structure and the morphological form of the novel verb must change? And, if so, does any advantage of RSR over repeated study continue to hold? At the 1-week mark, after the recall test using the original sentence structure, the children are presented with a task requiring them to use the novel verb in the structure *The Noun likes to Verb the Noun* (e.g., “This man likes to pum the frog”) where the novel verb must be used in bare-stem form rather than with the progressive –*ing* inflection.
3.  Finally, we ask if a similar recall and generalization pattern is seen in the two groups of children. In the Leonard et al. (2023) study, both groups showed better recall with RSR than with repeated study but the children with DLD had difficulty changing the morphological form of the novel verbs when it was required. A parallel finding would be seen in the present study if the children with DLD recalled the novel verbs but had trouble changing the syntactic structure or morphological form of the verb when it was needed. Such a finding would suggest that although children with DLD can learn new verbs in a sentence context (possibly helped by RSR), these verbs might not be sufficiently incorporated within the children's grammatical system.

## Method

This study was registered at ClinicalTrials.gov (Laurence B. Leonard, NCT06001866). The recruitment and experimental procedures used here were approved by the author's institutional review board. Written consent was obtained from the children's families, and verbal assent was provided by the children.

### Participants

Participants were 27 children, with 14 meeting the selection criteria for DLD and 13 meeting the criteria for TD. All were monolingual English speakers. The two groups were similar in age (DLD: *M* = 57.21 months, *SD =* 5.19; TD: *M* = 59.00 months, *SD* = 5.26) and socioeconomic status, as measured in years of maternal education (DLD: *M* = 16.71, *SD =* 3.36; TD: *M* = 17.42, *SD* = 2.36). For inclusion in the study, all children passed a hearing screening in both ears at 20 dB at 500, 1000, 2000, and 4000 Hz and scored above 75 on the Kaufman Assessment Battery for Children–Second Edition (KABC-2; Kaufman & Kaufman, 2004). A standard score of 75 on the KABC-2 falls above the level of intellectual disability even after the standard error of measurement is taken into account.

Nine girls and five boys comprised the DLD group. Ten children were White, two were Black, one was Asian, and one was of more than one race. These children were enrolled in a language intervention program or were already scheduled to begin such an intervention program. Each child in the DLD group was required to score below 87 on the Structured Photographic Expressive Language Test–Preschool 2 (SPELT-P2; Dawson et al., 2005), the cutoff exhibiting acceptable sensitivity and specificity (Greenslade et al., 2009). These children also met the criterion of scoring in the range of “minimal to no symptoms of autism spectrum disorder” on the Childhood Autism Rating Scale–Second Edition (CARS-2; Schopler et al., 2010).

The TD group consisted of seven girls and six boys. Eleven children were White, one was Black, and one was of multiple races. No problems with development in general or language in particular were reported for these children. They all met the language criterion of scoring above 87 on the SPELT-P2. They were not administered the CARS-2, as no child's developmental history suggested any concerns.

Additional measures were obtained from the children and their families for descriptive purposes; they were not part of the selection criteria. Two of these measures served as covariates, as in our previous studies—the standard score on the Peabody Picture Vocabulary Test–Fifth Edition (PPVT-5; Dunn, 2019) and maternal education in years. The third measure was the standard score of the Expressive Vocabulary Test–Third Edition (Williams, 2019). A summary of the scores on the criterion measures and descriptive measures can be seen in Table 1.

**Table 1.** Summary of the test scores and related information obtained from the children with developmental language disorder (DLD) and with typical language development (TD).

Variable	DLD (*n* = 14)	TD (*n* = 13)
Age in months	57.21 (5.19)	59.00 (5.26)
Sex	9 F, 5 M	7 F, 6 M
Maternal education in years^a^	16.71 (3.36)	17.42 (2.36)
SPELT-P2 (SS)^b^	75.93 (9.79)	117.92 (5.24)
KABC-2 (SS)^b^	104.29 (6.93)	107.92 (10.56)
PPVT-5 (SS)^a^	98.07 (11.28)	116.69 (10.98)
EVT-3 (SS)^c^	96.21 (10.64)	111.00 (11.73)

*Note.* F = female; M = male; SPELT-P2 = Structured Photographic Expressive Language Test–Preschool 2; SS = standard score; KABC-2 = Kaufman Assessment Battery for Children–Second Edition; PPVT-5 = Peabody Picture Vocabulary Test–Fifth Edition; EVT-3 = Expressive Vocabulary Test–Third Edition.

a Covariate measure.

b Selection criterion measure.

c Additional clinical measure.

One additional measure was obtained to assist in the scoring of the children's productions of the novel verbs during learning and on the recall tests. As in our prior studies, we employed a real word production task that focused on the initial consonants, vowels, and final consonants that were used for the novel verbs. For example, for the novel verb /tɛb/, actual words included “toy” and “web.” The children were presented with these words in short phrases and were asked to repeat them. In each case, the relevant real word appeared in sentence-final position (e.g., “Say ‘A spider has a web’”). Although the scoring procedure used for the novel verbs (see below) allowed for common developmental errors (e.g., /dit/ in place of /dik/), the production task alerted us to any unusual production patterns exhibited by the child that might complicate scoring.

A final measure was used to ensure that the children could consistently use the target syntactic structure that was to be employed during the learning period (*The Noun is Verb-ing the Noun*). In this task, toy characters were made to perform common actions such as reading a book and rolling a ball. The child and a “shy turtle” puppet were to observe the actions, but the shy turtle (with its head withdrawn into its shell) did not see, and the child was then asked to tell the shy turtle what the toy characters were doing (e.g., “Pooh is rolling the ball”). All children readily produced numerous utterances of the target structure.

### Materials

#### Novel Verbs, Sentences, and Actions

The same eight novel words used in Leonard et al.'s (2023) study were used as novel verbs in the present study. The novel verbs were divided into two sets of four. In each set, two novel words were assigned to the RSR condition, and two were assigned to the repeated study condition. Counterbalancing was used so that each novel word appeared in both conditions across children in each group. All novel words were consonant–vowel–consonant (CVC) monosyllables, specifically, /meɪp/, /faɪb/, /dik/, /gɪn/, /nok/, /jæd/, /pʌm/, and /tɛb/. Each novel word had an initial consonant and vowel that was unique to that word. Final consonants were shared but not for words in the same set. The novel words in the two conditions within the same set were matched according to average biphone frequency and neighborhood density based on Storkel's (2013) study.

The eight novel verbs were referents for eight novel transitive actions that were video-recorded using four different men and four different women as actors. For each novel verb, four video recordings were created to be used during the learning period, two with the same man performing the novel action on two different objects (in separate video clips) and two with the same woman performing the action on the same two objects (in separate video clips) as the man. An example appears in (1):

(1)

Woman 1 pums a toy frog

Man 1 pums a toy frog

Woman 1 pums a toy cow

Man 1 pums a toy cow

Additional video recordings of the novel actions were created as generalization items on the postlearning tests. New male and female actors were enlisted to record these items. For each novel verb, two video recordings were made, one with the original actor acting on a new object and one with a new actor acting on one of the original objects, as seen in (2):

(2)

Woman 1 pums a toy dog

Woman 2 pums a toy frog

The novel transitive actions that were used as referents for the eight novel word forms came from previously published studies (Horvath & Arunachalam, 2021; Imai et al., 2008; Ma et al., 2023), and five were created for this study. The actions were carried out by actors on common objects or toy stuffed animals. All object nouns were highly familiar to young children. The actions varied in appearance within and across sets, but all represented activity verbs. For example, one action (“yadding”) showed an actor sitting in a chair, facing sideways, with the object (a toy stuffed elephant) resting on his lower leg; he slowly and continuously kicked out his lower leg, raising and lowering the elephant. In another action (“tebbing”), the actor sat at a table with a large bowl of candy in front of her; she continuously lifted up handfuls of candy, letting the pieces slowly drop back into the bowl.

The novel actions were recorded on an iPhone in a well-lit room against a plain, dark blue background. In all cases, the actor was seated, facing sideways to demonstrate the action or facing forward at a table. Because each actor was used twice (acting out one verb for each set), they changed clothing and appearance (hairstyle, glasses) to make the actions more distinct and to create visual interest. During recording, the actions were performed to the timing of a metronome; this ensured a consistent pace across different actors and different actions. The recordings were edited to 10- to 12-s clips and combined with the audio-recorded stimuli using iMovie.

During each learning trial, the action was performed continuously while two audio-recorded sentences were presented in succession, as in (3). At the start of the trial, children viewed the action for 2 s, before the first sentence was heard. The action continued during 2.5 s of silence, after which the second sentence was heard. The action then continued in silence to the end. The same video clip that was used for the learning trials was used for recall and recognition, with different audio. As shown in (3), novel verbs were presented in progressive *–ing* form, preceded by the auxiliary *is*. The subject/agent of all sentences was either “this/that/the woman” or “this/that/the man.” The direct object/patient was the name of a toy stuffed animal or object preceded by an article. The sentences used appear in Appendix A. Note that in Leonard et al.'s (2023) study, each study trial included three repetitions of the sentence and thus an additional exposure to the novel verb relative to the present study.

(3)

The woman is pumming the cow. That woman is pumming the cow.

The sentences served as study trials for both the novel verbs in the RSR condition and for those in the repeated study condition. For retrieval trials (occurring only for novel verbs in the RSR condition), the same video clips were shown and the child was prompted to produce the appropriate sentence with the request, “Tell me what's happening here. The … ” or “What's happening here? The … .” The article was provided in the prompt to promote the child's use of the noun subject when responding.

Finally, to prepare the children for the novel verb learning task, we created both practice items and familiarization items. The three practice items featured actors performing a familiar action on a familiar object. The accompanying audio recording matched the sentence structure in which the novel verbs would be heard, as in, “The man is cutting the paper” and “The woman is bouncing the ball.” After each video and accompanying audio-recorded sentences was heard, the video re-appeared and the child was asked, “What's happening here? The … .” These three items served to alert the child that retrieval of the sentences would be involved.

The familiarization items introduced the children to the novel actions and accompanying novel verbs that they would see during learning. For each novel verb/action, two video clips were shown side by side, one with a woman performing the action on an object and the other with a man performing the action on a different object. The accompanying audio recording was of the form, “Look! They are pumming those animals” or “Wow! They are noking those things.” The specific names of the subjects/agents (such as man/woman) and toy animals and objects (such as dog/hat) were not used in these familiarization items. The children were not asked to retrieve these sentences.

#### Postlearning Tests

During the postlearning period, the children were administered three types of tests of their ability to recall, generalize, and recognize the novel verbs in sentences. The first test, referred to as “Verb Recall in Sentences,” consisted of 16 items and was designed to elicit the same syntactic structure as used in the learning period. Each novel verb was tested in four items. Two of the items required the children to recall the verb in sentences that were used during the learning period. As the video clip was shown, the child was asked, “What's happening here? The … .” The other two items of each novel verb required the child to recall the novel verb in sentences (of the same structure) with an original actor acting on a new object and a new actor acting on one of the original objects. Therefore, there were eight items requiring the child to recall the verb in sentences that had already been heard, referred to as “learned items,” and eight items requiring recall of the novel verbs in similar sentences but with a new subject/agent or object/patient. These are referred to as “generalization items.” This Verb Recall in Sentences test was administered 5 min after the second day of the learning period and 1 week later.

The second test administered was used only at the 1-week test point. In this task, the child was required to retrieve the novel verb in a new sentence structure after hearing a model sentence using this new structure with a familiar verb. This test is referred to as “Verb Recall in a New Structure” and took the form of a syntactic priming task. Each item consisted of a pair of video clips with a description of the first serving as a prime and the second, to be described by the child, representing the target. The test began with three practice items. The child saw a video of a woman or man performing a familiar action on a familiar object and heard a sentence of the structure *The Noun likes to Verb the Noun* (e.g., “This woman likes to catch the ball, but this … ”). A video was then shown depicting a different familiar action performed by a different actor on the object (e.g., a woman bouncing a ball), and the child was asked to describe it. After the three practice items, 16 items were presented. For each item, the first video described in the audio had the above structure using familiar verbs (e.g., “This man likes to swing the frog, but this … ”), and the second video—to be described by the child—depicted a different actor performing a novel action on the same object used in the prime (e.g., a different man pumming the frog). If priming was successful and the novel verb was retrieved, the child's production would resemble the target, “(This) man likes to pum the frog.” Four items were used for each novel verb. All items on this test required a syntactic structure that differed from the one used during the learning period.

The third test was also administered only at the 1-week test point. It is referred to as the “Verb Recognition” test, requiring the child to match the audio-presented novel verb to the appropriate video-recorded action. Two video clips were shown side by side. One practice item was used in which both video clips depicted familiar actions (a man reading a book and a man cutting paper). The child then heard a request to point to the correct picture in the form of, “Which one shows ‘the man is reading’?” Sixteen items were then presented involving two adjacent video clips each depicting a man or woman performing a novel action on an object (e.g., a man ginning a carrot, a man deeking a towel). The child was requested to point to the correct video (e.g., “Which one shows ‘the man is ginning’?”). Four items were used for each novel verb; two were learned items, and two were generalization items.

### Procedure

Details of the procedure appear in Appendix B. The two sets of four words were learned sequentially. One week after the final tests for the first set, learning began for the second set. In each set, the learning period began with the practice items and familiarization items. Items from both conditions were included in each set, presented in alternating order, with the specific order (beginning with RSR or with repeated study) counterbalanced across children in each group. Half the items in each condition featured a man performing the action, and half featured a woman performing the action.

The learning period for each set involved two 20-min sessions held on consecutive days. For novel verbs in the RSR condition, the first two retrieval trials were immediate retrieval trials. After viewing and listening to a study trial (e.g., “The woman is pumming the cow. That woman is pumming the cow”), the video was re-presented and the child was given the retrieval prompt (“What's happening here? The … ”). Thereafter, all retrieval trials for the novel verb appeared after items for the other three novel verbs had intervened. These were the spaced retrieval trials. All retrieval trials—immediate and spaced—were directly followed by another study trial for the same novel verb. The novel verbs in the repeated study condition received the same kinds of study trials but no retrieval trials. The second day was identical to the first day except that the order of the items was changed. Across the 2 days, there were 16 study trials for each novel verb and 12 retrieval trials (four immediate, eight spaced) for each novel verb in the RSR condition. The same number of study trials was provided for the novel verbs in the two conditions. Because two sentences with the novel verb were used in each study trial, the total number of exposures to each novel verb in the study trials was 32. (Recall that each novel verb was also heard twice on each day as part of familiarization, bringing the total number of exposures to 36.) Although not initially based on Storkel et al.'s (2019) study, we note that those investigators found that 36 exposures lead to significant word learning in children with DLD.

Five minutes after the second learning session, the Verb Recall in Sentences test was administered. The prompts for this 16-item test were the same used for the retrieval trials in the learning period (“What's happening here? The … ”). Each verb was tested with four items, two learned items and two generalization items. For this test, the order of verbs was again changed and presented in blocks of alternating item type (i.e., the four verbs were first tested using learned items and then generalized items, followed by another block of learned and then generalized items).

One week later, the Verb Recall in Sentences test was re-administered. We then presented the children with the Verb Recall in a New Structure test. After the three practice items involving familiar verbs, the 16 items requiring the children to use the novel verbs in the structure *The Noun likes to Verb the Noun* were presented. The remaining test—the Verb Recognition test—was then administered. There were four items per novel verb, half representing learned items and half representing generalization items.

### Scoring and Reliability

For the Verb Recall in Sentences test and the Verb Recall in a New Structure test, scoring criteria for the children's accuracy in recalling the novel verb forms were of major importance. However, the children's use of the target sentence structure also had to meet at least minimal standards.

The scoring of the children's productions of the word forms on the recall tests followed a multistep process. First, if the production represented a real word that might have been intended as an alternative name for the action (e.g., “kick”), it was scored as incorrect. Second, any productions that appeared to be a potential attempt at the novel verb were examined further. Productions that matched the adult pronunciation of the novel verb were scored as correct. Those that deviated from adult pronunciation were scored according to the system developed by Edwards et al. (2004). Consonants were scored with 1 point each for accuracy in place, manner, and voicing. Vowels were given 1 point each for length, height, and backness. An additional point was awarded for the correct CVC syllable shape. This resulted in a maximum of 10 points per word. If, for example, /gɪn/ was produced as /dɪn/, 1 point would be deducted for the place of articulation error in the initial consonant, resulting in a score of 9 (2 + 3 + 3 + 1). We then compared the resulting score to the score that would be assigned if we assumed the child was attempting one of the other novel verb forms. For example, if /dɪn/ were actually an attempt at /dik/, it would earn a total of only 6 points (3 + 2 + 0 + 1). Given the lower score (and others like it), we would assume that /dɪn/ was a reasonable attempt at /gɪn/ and would be scored as correct. This scoring method allowed for some phonetic imprecision yet permitted a correct/incorrect decision. An alternative of simply scoring each production on the 1–10 scale had the disadvantage of not distinguishing between bona fide attempts at the correct word form and productions that may not have even been attempts at the correct word.

Criteria for correct use of the target sentence structure in the Verb Recall in Sentences test were production of the subject/agent noun, the correct novel verb inflected with –*ing*, and the object/patient noun (e.g., “Woman pumming the cow”). Allowances were made if the children used the wrong noun if the error constituted a reasonable misidentification. Because many children with DLD in this age range are inconsistent in their use of auxiliary *is* (Leonard, 2014), the children's inclusion of this grammatical morpheme in their response was not required. For the sentence structure on the Verb Recall in a New Structure test to be scored as correct, the response had to include the subject/agent noun, the verb *like*, and the correct novel verb in bare-stem form (e.g., “Man like to pum the frog”). The grammatical morphemes present third-person singular –*s* and infinitival *to* are used inconsistently by children with DLD at this age (Leonard, 2014) and were therefore not required.

To assess scoring reliability, the responses of four children in each group were selected and scored independently by a second judge. Reliability was computed for both the 5-min and 1-week Verb Recall in Sentences tests as well as for the Verb Recall in a New Structure test administered at the 1-week mark. Correct responses were defined as recall of the correct novel verb based on the Edwards et al. (2004) system and produced within the sentence structure required for each test, as specified above. For the Verb Recall in Sentences tests, item-by-item interjudge reliability for correct–incorrect judgments was 100% for the both children with DLD and the children with TD. For the Verb Recall in a New Structure test, reliability was 100% for the DLD group and 96.88% for the TD group.

### Data Analysis

Mixed-effects models were used to evaluate the children's responses on the Verb Recall in Sentences test, the Verb Recall in a New Structure test, and the Verb Recognition test. Models were run with and without the covariates of PPVT-5 and maternal education in years. The number of items correct was the outcome measure for each analysis. The Verb Recall in Sentences Test was administered 5 min after the learning period and 1 week later. For this test, diagnostic group (DLD, TD) was a between-participants variable and learning condition (RSR, repeated study), time (5 min, 1 week), and item type (learned, generalized) were within-participants variables. The Verb Recognition test was administered only at the 1-week mark and therefore did not include time as a variable. The Verb Recall in a New Structure test, also administered only 1 week later, required the children to use a sentence structure not used during the learning period, so neither item type nor time was included in the analysis. We included random slopes for learning condition, item type, and time when they did not approach zero.

Main-effects models and full factorial models with all possible interactions were tested hierarchically. The main-effects models are presented initially with no interactions to provide a baseline of each model variable. Statistically significant interactions are then presented, and relevant simple effects are reported. Effect sizes are presented as partially standardized beta coefficients (*b*_std_). These are comparable to *d*, although they reflect conditional standardized mean differences conditioned on other variables in the model. To account for nonnormal error terms, we used bootstrapped standard errors with 1,000 replicates. Stata Version 18.0 (StataCorp, 2023) was used for the mixed-effects model analyses.

## Results

### Verb Recall in Sentences

We first analyzed the data related to children's ability to recall the novel verbs in the sentence structure used during learning. Main-effects models for the Verb Recall in Sentences test appear in Table 2. Random slopes for learning condition and time were included in the models. We focus on the results using covariates. Scores ranged from 0 to 8. A learning condition effect was seen with scores for RSR words 1.09 points higher than scores for the repeated study words, translating to a medium effect size *b*_std_ of 0.47. A small effect size (*b*_std_ = 0.16) was seen for time, with scores for the 1-week test 0.37 points higher than scores for the 5-min test. Neither diagnostic group nor item type was statistically reliable. A Learning Condition × Time interaction was observed (*p =* .028), with simple effects showing that the scores for RSR were higher than scores for repeated study at both 5 min (*b*_std_ = 0.60) and 1 week (*b*_std_ = 0.34), with comparable scores at 5 min and 1 week for the RSR words but higher scores at 1 week than at 5 min for repeated study words (*b*_std_ = 0.29).

**Table 2.** Verb Recall in Sentences: main-effects model results (*N* = 27, observations = 216).

Variable	Main effects: no covariates					Main effects: with covariates				
	*b*	95% CI		b*	*p*	*b*	95% CI		b*	*p*
Fixed effects										
Group (DLD vs. TD)	−0.56	−1.84	0.72	−0.24	.389	−0.98	−2.59	0.64	−0.42	.236
Condition (RSR vs. RS)	1.09	0.54	1.64	0.47	.000	1.09	0.55	1.63	0.47	.000
Item (learn vs gen)	0.09	−0.01	0.20	0.04	.091	0.09	−0.02	0.20	0.04	.101
Time (1 wk vs. 5 min)	0.37	0.07	0.67	0.16	.016	0.37	0.06	0.68	0.16	.019
Covariates										
PPVT						−0.03	−0.08	0.02	−0.01	.271
Mother's education						0.18	−0.07	0.43	0.08	.156
Intercept	3.41	2.34	4.48		.000	3.65	−3.42	10.71		.312
Random effects	σ^2^					σ^2^				
Condition	3.39	2.09	5.52			3.40	2.11	5.47		
Time	0.85	0.18	3.96			0.85	0.18	3.91		
Intercept	4.27	2.56	7.15			4.21	2.53	7.01		
Residual	0.41	0.27	0.64			0.41	0.27	0.63		

*Note. N* = 27, observations = 216 (we used bootstrapped standard errors with 1,000 replicates). Effects (*b*) with 95% confidence intervals (CIs) that do not include 0 are statistically significant at α = .05. *b* is the unstandardized coefficient; b* is the partially standardized coefficient where the outcome is in standard deviation units. σ^2^ is the estimated variance of the random effect. The fixed-effects intercept is the estimate mean of the outcome when all model covariates are zero. The random-effects intercept is the estimated between-participants variation in the outcome means conditioned on the model covariates. DLD = children with developmental language disorder; TD = age-matched children with typical language development; RSR = repeated spaced retrieval condition; RS = repeated study condition; learn vs. gen = learned vs. generalization; wk = week; PPVT = Peabody Picture Vocabulary Test–Fifth Edition.

**Figure 1.**
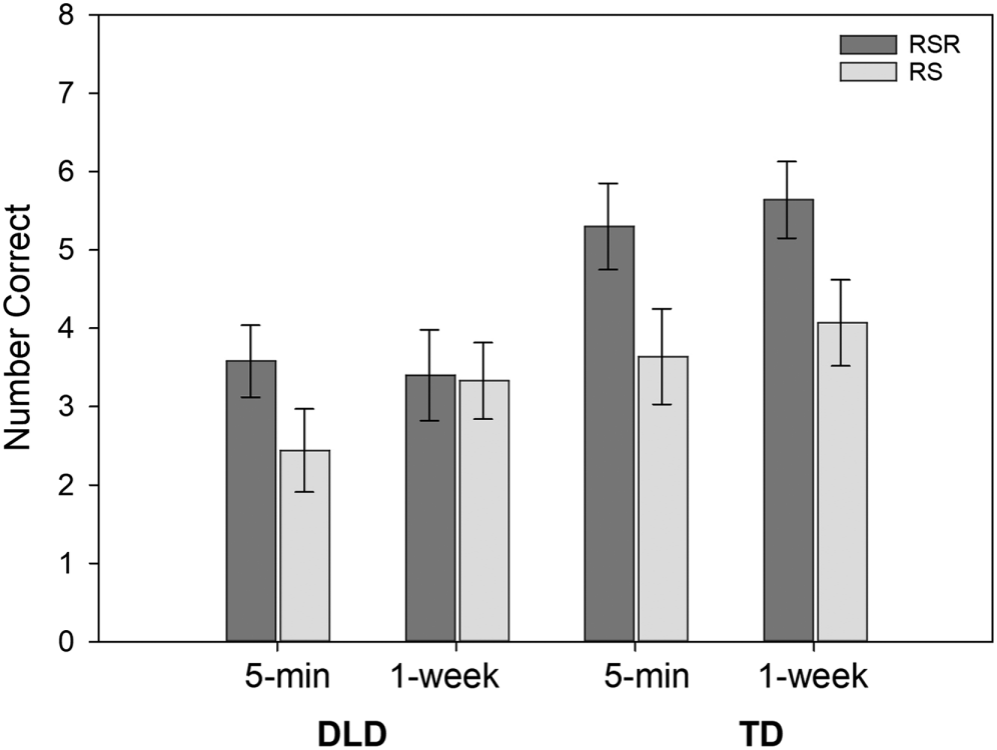
The conditional means and standard errors reflecting the number of novel verb items recalled correctly on the Verb Recall in Sentences test. RSR = repeated spaced retrieval condition; RS = repeated study condition; 5-min = number correct when tested 5 min after the learning period; 1-week = number correct when tested 1 week after the learning period; DLD = children with developmental language disorder; TD = children with typical language development.

The Learning Condition × Time interaction can be better understood through the three-way interaction of Diagnostic Group × Learning Condition × Time (*p* = .053). This interaction is illustrated in Figure 1. Key factors in this interaction were the findings that, for the TD group, scores were higher for RSR words than for repeated study words at both 5 min (1.65 points higher, *b*_std_ = 0.71) and 1 week (1.59 points higher, *b*_std_ = 0.68). However, for the children with DLD, the difference favoring RSR words (1.14 points higher, *b*_std_ = 0.49) held only for 5-min testing, owing primarily to a clear increase in scores for repeated study words from 5 min to 1 week. An additional finding was that differences favoring the TD group over the children with DLD applied only to the RSR condition (5 min: *b*_std_ = −0.74; 1 week: *b*_std_ = −0.96).

**Figure 2.**
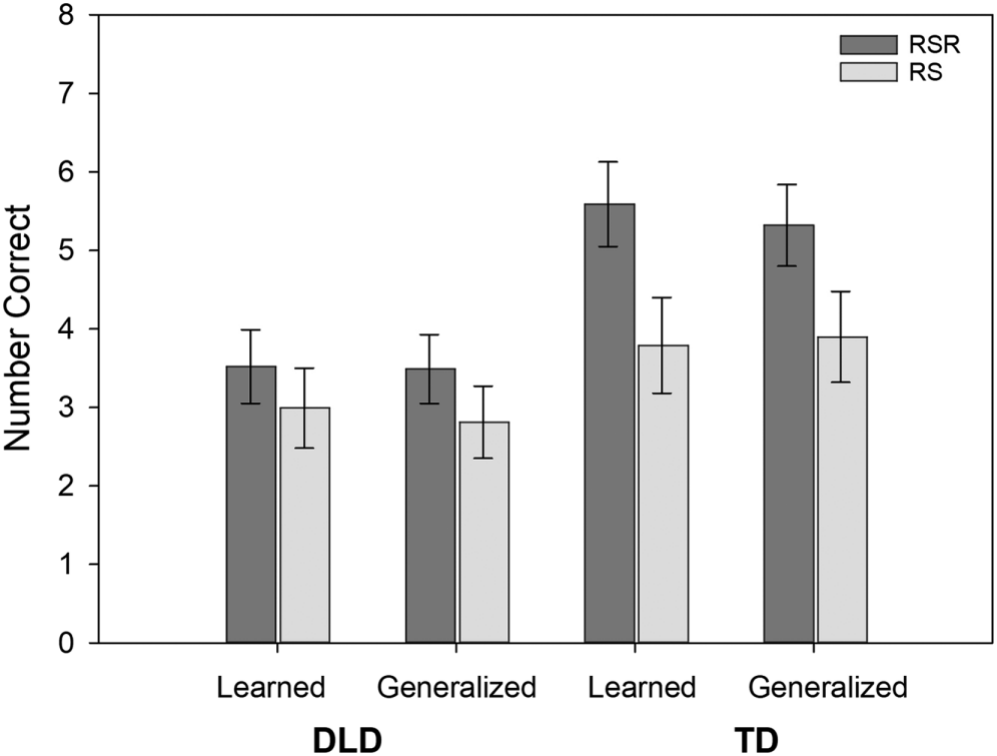
The conditional means and standard errors reflecting the number of novel verb items recalled correctly on the Verb Recall in Sentences test. RSR = repeated spaced retrieval condition; RS = repeated study condition; Learned = number of items recalled that required novel verb to be used in sentences that were included in the learning period; Generalized = number of items recalled that required novel verb to be used in sentences that were not included in the learning period; DLD = children with developmental language disorder; TD = children with typical language development.

Although item type showed no main effect once covariates were applied, it was involved in one interaction—the three-way interaction of Diagnostic Group × Learning Condition × Item Type (*p* = .030). Figure 2 provides an illustration of this interaction. Simple effects indicated that, for the TD group, scores were higher for the RSR condition than for repeated study for both learned items (1.81 points higher, *b*_std_ = 0.78) and generalization items (1.42 points higher, *b*_std_ = 0.61). However, for the children with DLD, the advantage for RSR words was only marginally better (0.68 points higher, *b*_std_ = 0.29) for generalization items and was no higher than the repeated study words for learned items. In addition, differences favoring the TD group over the DLD group were seen for the RSR condition for both learned items (2.07 points higher, *b*_std_ = −0.89) and generalization items (1.84 points higher, *b*_std_ = −0.79), but these differences did not hold for the repeated study condition.

### Verb Recall in a New Structure

**Figure 3.**
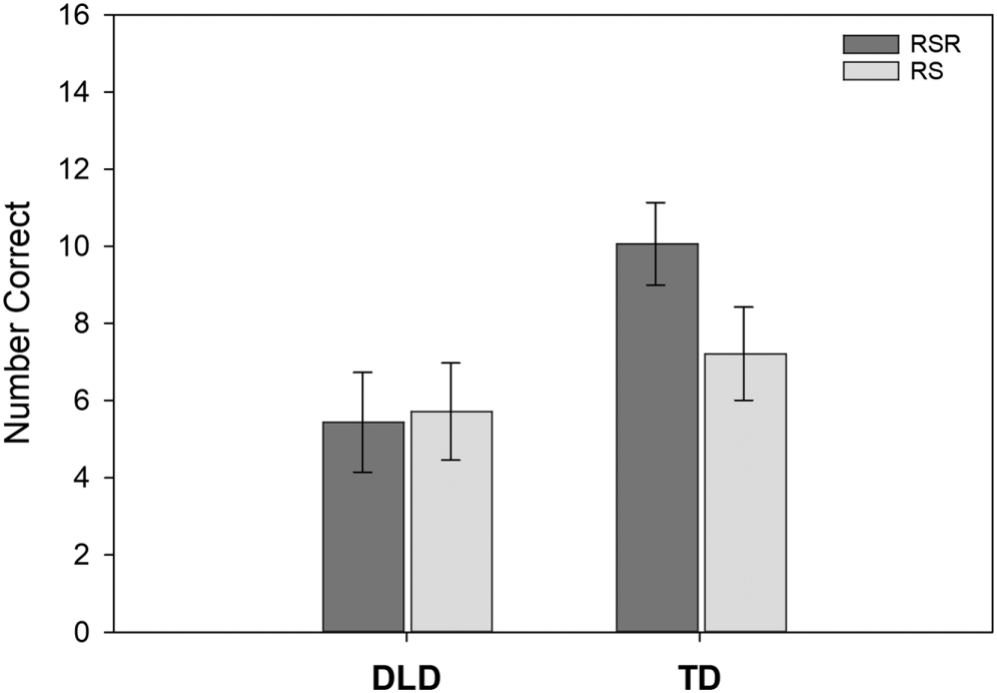
The conditional means and standard errors reflecting the number of novel verb items recalled correctly on the Verb Recall in a New Structure test. RSR = repeated spaced retrieval condition; RS = repeated study condition; DLD = children with developmental language disorder; TD = children with typical language development.

Children were also tested on their ability to produce the novel verbs in a new sentence structure—one different from what they had heard and used during learning. Table 3 shows the results for the main-effects models for the Verb Recall in a New Structure test. No random slopes were included in the models. Scores ranged from 0 to 16. The main effects for both diagnostic group (*p* = .075) and learning condition (*p* = .073) were marginal once covariates were applied. However, a Diagnostic Group × Learning Condition interaction was observed (*p* = .014). Simple effects revealed that, for the TD group, scores for the RSR condition were 2.85 points higher than scores for the repeated study condition (*b*_std_ = 0.55). However, there was no difference according to learning condition for the children with DLD. In addition, the TD group had higher scores than the children with DLD for novel verbs in the RSR condition (4.63 points higher, *b*_std_ = 0.89) but did not differ from the children with DLD for novel verbs in the repeated study condition. This interaction is shown in Figure 3.

**Table 3.** Verb Recall in a New Structure: main-effects model results (*N* = 27, observations = 54).

Variable	Main effects: no covariates					Main effects: with covariates				
	*b*	95% CI		b*	*p*	*b*	95% CI		b*	*p*
Fixed effects										
Group (DLD vs. TD)	−3.38	−6.27	−0.49	−0.65	.022	−3.06	−6.43	0.31	−0.59	.075
Condition (RSR vs. RS)	1.22	−0.08	2.52	0.24	.065	1.22	−0.12	2.56	0.24	.073
Covariates										
PPVT						−0.01	−0.12	0.11	0.00	.900
Mother's education						0.65	0.09	1.21	0.12	.024
Intercept	8.20	6.19	10.21		.000	−2.19	−17.80	13.43		.784
Random effects	σ^2^					σ^2^				
Intercept	18.41	10.22	33.18			16.39	9.27	28.97		
Residual	6.55	4.09	10.50			6.55	4.08	10.51		

*Note. N* = 27, observations = 54 (we used bootstrapped standard errors with 1,000 replicates). Effects (*b*) with 95% confidence intervals (CIs) that do not include 0 are statistically significant at α = .05. *b* is the unstandardized coefficient; b* is the partially standardized coefficient where the outcome is in standard deviation units. σ^2^ is the estimated variance of the random effect. The fixed-effects intercept is the estimate mean of the outcome when all model covariates are zero. The random-effects intercept is the estimated between-participants variation in the outcome means conditioned on the model covariates. DLD = children with developmental language disorder; TD = age-matched children with typical language development; RSR = repeated spaced retrieval condition; RS = repeated study condition; PPVT = Peabody Picture Vocabulary Test–Fifth Edition.

Contributing to the generally lower scores of the children with DLD was the fact that seven of the children in this group produced a total of 35 responses that maintained the –*ing* inflection on the novel verb instead of changing the novel verb to the bare-stem form (e.g., “This man likes to pumming the frog”). In some of these cases, the children also persisted with the original structure (e.g., “This man is pumming the frog”) instead of changing the structure called for (e.g., “This man likes to pum the frog”). Difficulties in applying the bare-stem form of the verb were seen for novel verbs in both conditions. Only a single instance of incorrect use of –*ing* with the novel verb was seen in the TD group, although one child with TD did produce several instances of a grammatically correct form with –*ing*, as in, “This man likes pumming the frog.” (No instances of this latter form were observed in the DLD data.)

### Verb Recognition

Finally, we examined the data related to children's accuracy in recognizing each novel action upon hearing the novel verb. No main effects for this test were observed. Ceiling effects were present, which greatly reduced variability in scores and, subsequently, statistical power to detect effects.

## Discussion

A goal of the present study was to determine whether RSR could assist children with DLD in learning novel verbs when these verbs appear in a transitive sentence structure and must be produced by the children with the same structure during the learning period. A previous study (Leonard et al., 2023) showed beneficial effects for RSR on novel verb learning, but scores overall were quite low. In that study, novel intransitive body movements were performed by actors and only single-word, bare-stem responses were required. For the current study, we used the design and specific novel word forms of the previous study but used actors performing actions on objects and required sentence-level responses containing the novel verb in transitive sentences (as in, “The woman is pumming the frog”). From the standpoint of improving children's overall scores with these changes, the current study appeared to be successful. For example, mean scores at 1 week for the children with DLD in Leonard et al.'s (2023) study were 2.40 and 1.01 for the RSR condition and repeated study conditions, respectively. In the present study, the corresponding scores were 3.40 and 3.33 for the children with DLD, respectively. Scores were also higher in the current study for the TD group relative to their counterparts in the earlier study. In the previous study, mean scores at 1 week were 3.45 and 2.07 for the RSR condition and repeated study conditions, respectively, compared to the present study with corresponding means of 5.64 and 4.07.

In the first part, we likened the procedure used during the learning period to a comprehension-to-production syntactic priming task (Bock et al., 2007) because the children were hearing a consistent syntactic structure. Although the sentences constituted a closed set of 16 specific sentences in each set, 5 min after the end of the learning period, the children were tested on sentences that included eight that differed from those used in the learning period by changing the subject/agent or object/patient. The fact that the children in both groups showed similar accuracy on these new sentences as on the previously heard sentences seems in line with the parallels between the children's learning experience and operations involved in syntactic priming (e.g., Rowland et al., 2012).

Unlike typical syntactic priming tasks, our procedure required the children to retrieve newly introduced verbs and incorporate them into the consistent syntactic structure. What success the children had in integrating the new verbs with the syntactic structure we attribute in part to the richer semantic and propositional information provided by the transitive structure and variation in subjects/agents and objects/patients within that structure.

As in the earlier study, we observed a main effect for learning condition showing an advantage for RSR over repeated study. Less expected was a Learning Condition × Time interaction showing that, although the RSR advantage held across both time periods, the difference was smaller (although still statistically reliable) at 1 week.

We were somewhat surprised that the main effect for diagnostic group was not statistically reliable despite the numerical advantages in scores seen for the children with TD. In general, children with TD are stronger word learners than children with DLD. In the previous novel verb study by Leonard et al. (2023), group differences favoring the TD group no longer held when standardized receptive vocabulary scores were applied as a covariate. Yet, in the current study, a main effect did not emerge even before applying the covariates. As discussed below, however, diagnostic group did play a role in particular interactions.

A common finding in the literature is for the RSR advantage over repeated study to remain stable over time for both children with DLD and their typical peers (Gordon et al., 2021; Leonard, Deevy, et al., 2019). However, in the current study, there was an unexpected interaction, caused principally by an increase in the repeated study scores of the children with DLD from 5-min testing to 1-week testing. In hindsight, the direction of this rather selective change has a potential explanation. In repeated study, the children heard the sentences throughout the learning period but did not have an occasion to produce them until the 5-min postlearning test. For the DLD group, in particular, producing these sentences at this point might have been a challenge. However, once having the knowledge that these sentences had to be produced, and gaining what experience they could garner over the eight repeated study items on the 5-min test, the children were better prepared for these items on the 1-week test. Because the verbs in the RSR condition had already been practiced in sentences during the learning period, the requirement to produce these verbs in sentences on the 5-min test constituted a less abrupt change. For the TD group, the requirement to produce the verbs in sentences in the repeated study condition on the 5-min test was apparently not a factor. The practice received on the RSR items during the learning period was sufficient.

Evidence supporting this speculation about the children with DLD comes from the previous Leonard et al. (2023) study. In that study, retrieval responses on the postlearning tests were at the one-word level (e.g., Experimenter: “Tell me about the woman. The woman likes to … .” Child: “teb”). Neither group showed any increase in scores from the 5-min test to the 1-week test in either the RSR or repeated study condition.

This potential suppression of correct responses at 5 min for the repeated study words by the DLD group was relative to their RSR words at 5 min and the repeated study words for the TD group at 5 min. On an absolute basis, the DLD scores for repeated study words at 5 min (*M* = 2.44) were nevertheless higher than what we found in the previous novel verb study that required only single-word responses (*M* = 1.26). In that study, scores for repeated study words were low at 5 min (*M* = 1.26) and remained low at 1 week (*M* = 1.01). Our tentative conclusion is that learning the novel verbs in sentences was beneficial for both conditions at both time points, but sentence-level responses on novel verb items that had not been practiced constitute more of a burden on children with DLD.

There is also a plausible explanation for the finding that the learning condition effect for the DLD group was weaker for learned items than for generalization items. As noted in the Method section, the first half of the learned items was presented before the first half of the generalization items; the same order of learned-before generalization items held true for the remaining items on the test. This provided the children with an earlier opportunity to practice using the novel verbs in sentences representing learned items, including words from the repeated study condition. The TD group, in contrast, showed the expected results of a clear advantage of RSR over repeated study for both learned and generalized items.

The findings for the Verb Recall in a New Structure test were very informative. The children with TD were more successful using the correct novel verb in a new structure for RSR words than for repeated study words. For the children with DLD, the two conditions did not differ. Half of the children with DLD differed notably from their peers with TD in failing to produce the bare stem of the novel verb after hearing model sentences with real verbs that exemplified that form. Often, these errors occurred within an otherwise correct *likes to* structure, as in, “This man likes to pumming the frog,” although instances of failing to alter the syntactic structure used during the learning period (e.g., “This man is pumming the frog”) were also seen. The children with DLD were not observed to use a grammatical alternative that preserved –*ing* as in, “This man likes pumming the frog.” In general, these observations are consistent with findings that children with DLD have difficulties varying their morphosyntax if they have received considerable exposure to one particular morphosyntactic structure (see review in Leonard et al., 2024).

The specific difficulty with altering the morphological form of a new verb echoes a finding in the earlier Leonard et al. (2023) study. In that study, children learned the novel verbs as single-word bare-stem responses but, 1 week later, were given a task requiring them to use the novel verb in short intransitive sentences with progressive –*ing* (e.g., “The man is pumming”). These children had much more difficulty than their peers in producing the novel verb with –*ing* even though they had demonstrated frequent use of this inflection with familiar verbs. In the present study, problems occurred in the opposite direction—omitting –*ing* from the stem when the syntactic context required it. The present study joins the previous study, then, in identifying less morphosyntactic flexibility by children with DLD in their learning of new verb forms. Even when they have a degree of success in learning new verbs in sentences, these children may be too dependent on the syntactic structure and particular morphological form of the verb to readily use the verb when the original context changes.

We should emphasize that the children with DLD were able to benefit from the semantic richness of having an argument structure with different subjects/agents and objects/patients when they learned the novel verbs. This seems clear from the higher scores obtained in the present study relative to the earlier study, even given one-third fewer exposures to the novel verbs during learning in the present study (two exposures rather than three during each study trial). The obstacle occurred when a different structure and morphological form of the verb was needed.

This finding has clinical implications. These children might learn new verbs more easily in sentence contexts with some degree of semantic variety. However, clinicians should ensure that the children can demonstrate flexible use of these verbs both syntactically and morphologically before assuming that the children have acquired the verbs well enough to use them in their daily lives. This issue seems all the more important considering that the frequently seen benefits of RSR for word learning may not remain when children with DLD must alter the morphosyntax of their sentences. The contrast between the TD group and the DLD group in Figure 3 is striking in this regard. An informative study in the future might involve a comparison between RSR and repeated study conditions when novel verbs in both conditions are taught in a variety of syntactic and morphological contexts from the beginning rather than assessing the children's ability to make modification only after the learning period.

## Summary

In this study, we asked if novel verb learning and retention might be accomplished when it occurs within a rich sentence context and, if so, whether RSR would show the same advantages over repeated study seen in earlier investigations. For children with TD, this was decidedly the case. These children showed higher scores for both RSR and repeated study conditions relative to an earlier study requiring only single-word responses, yet RSR remained superior for recall both shortly after learning and 1 week later. These children could also readily use the novel verbs in (never-before-heard) uninflected form in a new sentence structure when provided models of the structure with familiar verbs. While doing so, they maintained the same RSR advantage over repeated study.

For the children with DLD, a more complex picture emerged. The clearest evidence for an advantage for RSR came at 5-min testing after the learning period. One week later, the difference evaporated thanks in part to an unexpected increase in scores for items in the repeated study condition. We suspect that this increase can be attributed to the children with DLD requiring practice producing those particular novel verbs in sentences. They gained some experience with this during the 5-min testing, which might have better prepared them for testing 1 week later. Note that, at 5 min, these children could use novel verbs from the RSR condition in sentences—even those with new subjects/agents and objects/patients—but these novel verbs had been produced in other similar sentences during the learning period, unlike the novel verbs assigned to the repeated study condition.

The difficulty experienced by the children with DLD in using novel verbs that they had heard but not produced in sentences is one side of the coin. The other is the difficulty they experienced after using verbs in one type of sentence and then having to use them in a different morphological form in a different sentence structure. These dual findings illustrate the delicate balance between these children's verb learning challenges and their difficulties with syntax and morphology. When new verbs meet structure, obstacles are compounded. It appears that when we assist these children in acquiring and using new verbs, we will need to be deliberative, carefully ensuring the children have sufficient practice with the verbs and can demonstrate flexibility in the morphological form of the verbs and the structures in which they appear. RSR appears helpful in assisting children with DLD in the learning and recall of new verbs in well-practiced sentences, even in using the verbs successfully when subjects/agents and objects/patients are changed during testing. However, when new structures and morphological forms of the verbs are required, we thus far see a clear added benefit of RSR only in children with TD. To further assist children with DLD, we should seek additional ways of promoting greater morphological and syntactic flexibility when introducing the children to new verbs. As seen in the present study, this level of flexibility requires more than successful verb recall.

## Data Availability Statement

The data sets used for this study are available from the corresponding author on reasonable request.

## Appendix A

### The Sentences Used During Study Trials

Set 1 Learning

The man is maping a lion. That man is maping the lion!

Now, the woman is ginning a spoon. That woman is ginning the spoon.

This woman is deeking a shoe. The woman is deeking the shoe.

This man is faibing a bird. The man is faibing the bird.

Now, the woman is maping the lion. That woman is maping the lion.

The man is ginning the spoon. That man is ginning the spoon!

Oh, the man is deeking the shoe. That man is deeking the shoe.

The woman is faibing the bird. That woman is faibing the bird.

Now the man is maping a deer. That man is maping the deer.

This woman is ginning the corn. The woman is ginning the corn!

This woman is deeking a towel. The woman is deeking the towel.

This man is faibing a turtle. The man is faibing the turtle.

Now the woman is maping the deer. That woman is maping the deer.

The man is ginning the corn. That man is ginning the corn.

This man is deeking the towel. The man is deeking the towel.

This woman is faibing the turtle. The woman is faibing the turtle!

Set 2 Learning

This woman is noking a hat. The woman is noking the hat.

Now, the woman is yadding a bear. That woman is yadding the bear.

The man is pumming a frog. That man is pumming the frog!

This man is tebbing the candy. The man is tebbing the candy.

Oh, the man is noking the hat. That man is noking the hat.

The man is yadding the bear. That man is yadding the bear.

The woman is pumming the frog. That woman is pumming the frog.

The woman is tebbing the candy. That woman is tebbing the candy!

This woman is noking a doll. That woman is noking the doll.

This woman is yadding an elephant. That woman is yadding the elephant!

Now, the man is pumming a cow. That man is pumming the cow.

This man is tebbing the money. The man is tebbing the money!

This man is noking the doll. That man is noking the doll.

The man is yadding the elephant. That man is yadding the elephant.

Now the woman is pumming the cow. That woman is pumming the cow.

This woman is tebbing the money. That woman is tebbing the money!

## Appendix B

### The Procedure Used During the Learning Phase and Postlearning Tests for One of the Two Sets of Four Novel Verbs

**Set 1, Day 1**

- Familiarization
      Familiarization trial: Study and retrieval of familiar verbs in sentences (*cut paper*, *bounce ball*, *read book*)
      Introduction of novel verbs /mep/, /gɪn/, /dik/, /fɑɪb/, and corresponding actions
- Learning phase

**Table:**

Subject	Verb	Object	Condition	Exposure
*Block 1*				
man_1_	/mep/	lion	RSR	Study–retrieval–study
woman_1_	/gɪn/	spoon	RS	Study–study
woman_2_	/dik/	shoe	RSR	Study–retrieval–study
man_2_	/fɑɪb/	bird	RS	Study–study
woman_3_	/mep/	lion	RSR	Study–retrieval–study
man_3_	/gɪn/	spoon	RS	Study–study
man_4_	/dik/	shoe	RSR	Study–retrieval–study
woman_4_	/fɑɪb/	bird	RS	Study–study
man_1_	/mep/	deer	RSR	Retrieval–study
woman_1_	/gɪn/	corn	RS	Study
woman_2_	/dik/	towel	RSR	Retrieval–study
man_2_	/fɑɪb/	turtle	RS	Study
*Block 2*				
woman_3_	/mep/	deer	RSR	Retrieval–study
man_3_	/gɪn/	corn	RS	Study
man_4_	/dik/	towel	RSR	Retrieval–study
woman_4_	/fɑɪb/	turtle	RS	Study
man_1_	/mep/	lion	RSR	Retrieval–study
woman_1_	/gɪn/	spoon	RS	Study
woman_2_	/dik/	shoe	RSR	Retrieval–study
man_2_	/fɑɪb/	bird	RS	Study
woman_3_	/mep/	lion	RSR	Retrieval–study
man_3_	/gɪn/	spoon	RS	Study
man_4_	/dik/	shoe	RSR	Retrieval–study
woman_4_	/fɑɪb/	bird	RS	Study

**Set 1, Day 2**

- Familiarization
      (Re)Familiarization trial: Study and retrieval of familiar verbs in sentences (*cut paper*, *bounce ball*, *read book*)
      (Re)Introduction of novel verbs /mep/, /gɪn/, /dik/, /fɑɪb/, and corresponding actions
- Learning phase

**Table:**

Subject	Verb		Condition	Exposure
*Block 3*				
man_2_	/fɑɪb/	turtle	RS	Study–study
woman_2_	/dik/	towel	RSR	Study–retrieval–study
woman_1_	/gɪn/	corn	RS	Study–study
man_1_	/mep/	deer	RSR	Study–retrieval–study
woman_4_	/fɑɪb/	turtle	RS	Study–study
man_4_	/dik/	towel	RSR	Study–retrieval–study
man_3_	/gɪn/	corn	RS	Study–study
woman_3_	/mep/	deer	RSR	Study–retrieval–study
man_2_	/fɑɪb/	bird	RS	Study
woman_2_	/dik/	shoe	RSR	Retrieval–study
woman_1_	/gɪn/	spoon	RS	Study
man_1_	/mep/	lion	RSR	Retrieval–study
*Block 4*				
woman_2_	/fɑɪb/	bird	RS	Study
man_2_	/dik/	shoe	RSR	Retrieval–study
man_1_	/gɪn/	spoon	RS	Study
woman_1_	/mep/	lion	RSR	Retrieval–study
man_2_	/fɑɪb/	turtle	RS	Study
woman_2_	/dik/	towel	RSR	Retrieval–study
woman_1_	/gɪn/	corn	RS	Study
man_1_	/mep/	deer	RSR	Retrieval–study
woman_4_	/fɑɪb/	turtle	RS	Study
man_4_	/dik/	towel	RSR	Retrieval–study
man_3_	/gɪn/	corn	RS	Study
woman_3_	/mep/	deer	RSR	Retrieval–study

III.  5-min break
IV.  Verb Recall in Sentences test
      Practice trials: Retrieval of familiar verbs in sentences (*cut paper*, *bounce ball*, *read book*)

**Table:**

Subject	Verb	Object	Condition	Item type
man_4_	/dik/	shoe	RSR	Learned
woman_4_	/fɑɪb/	bird	RS	Learned
woman_3_	/mep/	lion	RSR	Learned
man_3_	/gɪn/	spoon	RS	Learned
woman_2_	/dik/	cup	RSR	Generalized object
man_2_	/fɑɪb/	pig	RS	Generalized object
man_5_	/mep/	lion	RSR	Generalized subject
woman_5_	/gɪn/	spoon	RS	Generalized subject
woman_2_	/dik/	towel	RSR	Learned
man_2_	/fɑɪb/	turtle	RS	Learned
man_1_	/mep/	deer	RSR	Learned
woman_1_	/gɪn/	corn	RS	Learned
man_6_	/dik/	towel	RSR	Generalized subject
woman_6_	/fɑɪb/	turtle	RS	Generalized subject
woman_3_	/mep/	horse	RSR	Generalized object
man_3_	/gɪn/	carrot	RS	Generalized object

**Set 1, One week later**

V.  Repeat Verb Recall in Sentences test (see Day 2, IV)
VI.  Verb Recall in a New Structure test
      Practice trials: Primes and targets using familiar verbs (*tear/cut paper*, *catch/bounce ball*, *pour/drink juice)*

**Table:**

Prime	Target	Condition
This woman likes to DROP the corn, but …	this woman likes to GINN the corn.	RS
This man likes to HUG the lion, but …	this man likes to MAPE the lion.	RSR
This woman likes to KISS the pig, but …	this woman likes to FAIB the pig.	RS
This man likes to HOLD the towel, but …	this man likes to DEEK the towel.	RSR
This woman likes to HUG the horse, but	this woman likes to MAPE the horse.	RSR
This man likes to KISS the turtle, but …	this man likes to FAIB the turtle.	RS
This woman likes to DROP the spoon, but …	this woman likes to GINN the spoon.	RS
This man likes to HOLD the cup, but …	this man likes to DEEK the cup.	RSR
This man likes to KISS the bird, but this …	this man likes to FAIB the bird.	RS
This man likes to HUG the deer, but …	this man likes to MAPE the deer.	RSR
This woman likes to HOLD the towel, but …	this woman likes to DEEK the towel.	RSR
This man likes to DROP the carrot, but …	this man likes to GINN the carrot.	RS
This woman likes to KISS the turtle, but …	this woman likes to FAIB the turtle.	RS
This woman likes to HOLD the shoe, but …	this woman likes to DEEK the shoe.	RSR
This man likes to DROP the spoon, but …	this man likes to GINN the spoon.	RS
This woman likes to HUG the lion, but …	this woman likes to MAPE the lion.	RSR

VII.  Verb Recognition test

**Table:**

		Condition	Item type
**man**_**6**_ **/dik/ towel**	man_1_ /mep/ horse	RSR	Generalized subject
woman_3_ /mep/ lion	**woman**_**1**_ **/gɪn/ corn**	RS	Learned
**man**_**1**_ **/mep/ deer**	man_3_ /gɪn/ spoon	RSR	Learned
**man**_**2**_ **/fɑɪb/ pig**	man_5_ /mep/ lion	RS	Generalized object
woman_2_ /dik/ towel	**woman**_**3**_ **/mep/ lion**	RS	Learned
**man**_**3**_ **/gɪn/ spoon**	man_2_ /fɑɪb/ turtle	RS	Learned
woman_5_ /gɪn/ spoon	**woman**_**2**_ **/dik/ cup**	RSR	Generalized object
woman_1_ /gɪn/ carrot	**woman**_**6**_ **/fɑɪb/ turtle**	RS	Generalized subject
woman_6_ /fɑɪb/ turtle	**woman**_**3**_ **/mep/ horse**	RSR	Generalized object
**man**_**2**_ **/fɑɪb/ bird**	man_3_ /gɪn/ corn	RS	Learned
**woman**_**2**_ **/dik/ shoe**	woman_3_ /mep/ deer	RSR	Learned
woman_4_ /fɑɪb/ pig	**woman**_**5**_ **/gɪn/ spoon**	RS	Generalized subject
man_2_ /fɑɪb/ bird	**man**_**4**_ **/dik/ towel**	RSR	Learned
**man**_**3**_ **/gɪn/ carrot**	man_6_ /dik/ towel	RS	Generalized object
**man**_**5**_ **/mep/ lion**	man_4_ /dik/ cup	RSR	Generalized subject
woman_2_ /dik/ shoe	**woman**_**4**_ **/fɑɪb/ turtle**	RS	Learned

*Note.* Distinct male and female actors are designated by distinct subscripts. The target for each recognition task item is shown in bold font; the remaining entries are the foils. Condition and item type of target are shown. RSR = repeated spaced retrieval condition; RS = repeated study condition.
